# A pilot study using unique targeted testing of the urogenital microbiome has potential as a predictive test during IVF for implantation outcome

**DOI:** 10.1007/s00404-023-06987-w

**Published:** 2023-03-11

**Authors:** Gloria E. Evans, Vishakha Mahajan, Sarah Wakeman, Tania Slatter, Anna P. Ponnampalam, Trevor P. Anderson, Makhdoom Sarwar, John J. Evans

**Affiliations:** 1 Department of Obstetrics and Gynaecology, University of Auckland, 85 Park Road, Grafton, Auckland, 1023 New Zealand; 2 Liggins Institute, University of Auckland, Auckland, New Zealand; 3 Fertility Associates, Christchurch, New Zealand; 4 Department of Pathology, University of Otago, Dunedin, New Zealand; 5 Canterbury Health Laboratories, Christchurch, New Zealand; 6 Department of Obstetrics and Gynaecology, University of Otago, Christchurch, New Zealand

**Keywords:** IVF, Urogenital microbiome, Predictive test, Implantation rates

## Abstract

**Purpose:**

This pilot study aimed to develop a methodology characterising the urogenital microbiome as a predictive test in the IVF workup.

**Methods:**

Using unique custom qPCRs, we tested for the presence of specific microbial species from vaginal samples and First Catch Urines from the male. The test panel included a range of potential urogenital pathogens, STIs, ‘favourable bacteria’ (*Lactobacillus* spp.) and ‘unfavourable bacteria’ (anaerobes) reported to influence implantation rates. We tested couples attending Fertility Associates, Christchurch, New Zealand for their first round of IVF.

**Results:**

We found that some microbial species affected implantation. The qPCR result was interpreted qualitatively using the Z proportionality test. Samples from women at the time of Embryo Transfer who did not achieve implantation had significantly higher percent of samples that were positive for *Prevotella bivia* and *Staphylococcus aureus* compared to women who did achieve implantation.

**Discussion:**

The results provide evidence that most other microbial species chosen for testing had little functional effect on implantation rates. The addition of further microbial targets (yet to be determined) could be combined in this predictive test for vaginal preparedness on the day of embryo transfer. This methodology has a substantial advantage of being affordable and easily performed in any routine molecular laboratory. This methodology is most suitable as a foundation on which to develop a timely test of microbiome profiling. Using the indicators detected to have a significant influence, these results can be extrapolated.

**Conclusion:**

Using a rapid antigen test, a woman can self-sample prior to embryo transfer and obtain an indication of microbial species present which could influence implantation outcome.

## What does this study add to the clinical work

**Table:** 

This pilot presents a unique, practical methodology for predicting the effective urogenital microbiome in couples undergoing IVF.	

## Introduction

Lack of implantation of an embryo into the endometrium in women undergoing IVF is far too common an occurrence. Implantation rates worldwide are still disappointingly low [1] and few reliable predictive tests are available.

Researching the urogenital microbiome using next-generation sequencing (NGS) of the 16S ribosomal RNA gene has been reported [2]. The major findings were that women whose vagina was dominated by a variety of *Lactobacillus* species had the most favourable pregnancy outcome [2] and is still considered an indicator of vaginal health [3] by maintaining an acidic pH to aid protection from the effect of anaerobes and *Escherichia coli* [4].

Further, women who harboured anaerobes notably *Gardnerella vaginalis* [5, 6] causing bacterial/anaerobic vaginosis and potential pathogens *Enterococcus *sp*., Escherichia coli* and/or *Streptococcus *sp*.* also had a less favourable outcome [2, 3]. Similar findings are supported by others, in women [7–10] and in the male [11]. The vaginal microbiome has the potential to influence the conditions in the uterine cavity where implantation occurs in the uterine luminal epithelium, since vaginal bacteria can ascend into the uterine cavity [3]. Nevertheless, it has been reported that the microbiomes in the uterus and vaginal tract differ [12].

Because of the difficulty with recruitment, we report this preliminary data as a pilot study. This study investigated the concept that a range of specific microbial species present in the urogenital tract of couples undergoing their first round of IVF could affect the implantation rates of the transferred embryo.

This project determined to understand the biodiversity of the microbiome reported to influence IVF outcome. Our aim was to initiate the development of a rapid, affordable, predictive test of microbiome profiling in the routine IVF workup. Individual microbiome profiling could have the potential to assist the couple in deciding whether to continue IVF in a particular cycle.

Our testing panel included *Lactobacillus* species, anaerobes, potential urogenital pathogens and STIs. Additional testing also included the most common STI, *Human Papilloma Virus* (HPV). So far, 18 high-risk and 12 low-risk subtypes of genital HPV have been identified [13]. HPV has been implicated in influencing IVF outcome in women [14, 15] and in the male [16] although others have reported this association to be less clear [17]. Of the STIs included for testing *Ureaplasma urealyticum, Ureaplasma parvum* and *Mycoplasma hominis* have been reported to contribute to the condition of anaerobic vaginosis [4, 18] and been implicated in affecting reproduction outcomes [19, 20]. Further, *Mycoplasma genitalium* has also been reported in couples experiencing failed IVF [21].

## Methods

This study received the approval of the Southern Health and Disability Ethics Committee, New Zealand application number 15/STH/65.

### Sample population

Samples were collected prospectively from a heterogenous group of 32 couples who attended Fertility Associates clinic in Christchurch, New Zealand and satisfied the following criteria.

Inclusion criteria included: couples aged 20–40 without any confounding health issues, who had not taken antibiotics in the previous month, were non-smokers and who were undergoing their first, fresh IVF cycle.

Exclusion criteria included: women on frozen embryo transfer cycles. Male partners with frozen semen.

### Samples collected

Women had two vaginal samples collected, the first obtained by self-collection and the second collected by the clinician [22]:

1. Baseline Sample A, collected in the cycle before the IVF cycle, in the mid-luteal phase.
2. Sample B at fresh embryo transfer (ET).

and

3. The potential influence of the male partner was also explored collecting a baseline Sample C, either, fresh semen or a First Catch Urine (FCU). A further semen sample was collected at ET in MycoDuo media for *Mycoplasma* spp. culture.

Vaginal samples were collected repurposing swabs for molecular testing using the BD ProbeTec Qx collection kit 441357, Cat. 22-370-171 (Fisher Scientific).

Of the 32 couples who consented to participate in this project, 2 were excluded as they did not proceed on to IVF the following cycle. Therefore, *n* = 30 couples.

### Techniques employed for the detection of microbial organisms

1. Nugent Gram stain scores were assessed for visible bacterial populations present in vaginal swabs A and B [23].
2. qPCR for molecular presence or absence of microbial species in samples A, B and C [24].
3. Molecular detection for the presence or absence of *Human Papilloma Virus* (HPV)—with subtyping, using the EUROIMMUN HPV typing array (Perkin Elmer) [13] in samples A, B C or semen in MycoDuo media (ET).
4. Culture of male partner’s semen sample (at ET) collected in MycoDuo medium (Bio-Rad) [25] for the detection of *Mycoplasma hominis, Ureaplasma urealyticum* and *Ureaplasma parvum.*

#### Nugent Gram stain scores obtained from vaginal swabs

Gram staining was performed on all vaginal swabs. WBC, *Lactobacilli*, *Gardnerella* and anaerobes were noted [23]. Squamous epithelial cells were noted to confirm the sample had been well collected. Scores were allocated from 0 to 4 where 0 indicated no anaerobes present but a predominance of *Lactobacilli*, whereas a value of 4 indicated a predominance of *Gardnerella vaginalis* ± other anaerobes.

#### Molecular testing platform for microbial species

Unique Qiagen, Custom Microbial DNA qPCR Arrays Cat No. 330161 CBAID00051 (Qiagen) [24] were a user-defined assay developed specifically for this project. The PCR detected the bacterial 16S rRNA gene. Probes were designed for specific targets that were user-defined microbial species (Table 1).

**Table 1:** Microbial targets chosen for qPCR testing

Gene symbol	Target name for microbial species	NCBI taxonomy ID
A.Prev	*Anaerococcus prevotii*	33,034
C.Trac	*Chlamydia trachomatis*	813
E.Faecalis	*Enterococcus faecalis*	1351
F.Magn	*Finegoldia magna*	1260
G.Vagi	*Gardnerella vaginalis*	2702
L.Cris	*Lactobacillus crispatus*	47,770
L.Gass	*Lactobacillus gasseri*	1596
L.Iner	*Lactobacillus iners*	147,802
L.Jens	*Lactobacillus jensenii*	109,790
M.Geni	*Mycoplasma genitalium*	2097
M.Homi	*Mycoplasma hominis*	2098
N.Gono	*Neisseria gonorrhoeae*	485
P.Bivi	*Prevotella bivia*	28,125
S.Aure	*Staphylococcus aureus*	1280
S.Agal	*Streptococcus agalactiae*	1311
T.Vagi	*Trichomonas vaginalis*	5722
U.Parv	*Ureaplasma parvum*	2130
U.Urea	*Ureaplasma urealyticum*	1352
E.Faecium	*Enterococcus faecium*	1352
E.Coli	*Escherichia coli*	623
S.Pyog	*Streptococcus pyogenes*	1314
GAPDH	Hs GAPDH	
Pan1	Pan Bacteria 1	
PPC	Positive PCR Control	

The molecular testing platform was categorised into 5 groups:

1. Anaerobes—*Anaerococcus prevotii, Finegoldia magna, Gardnerella vaginalis* (facultative anaerobe)*, Prevotella bivia.*
2. *Lactobacillus* spp.—*Lactobacillus crispatus, Lactobacillus gasseri, Lactobacillus iners, Lactobacillus jensenii.*
3. Potential urogenital pathogens—*Enterococcus faecalis, Enterococcus faecium, Escherichia coli, Staphylococcus aureus, Streptococcus agalactiae, Streptococcus pyogenes*.
4. STIs—*Chlamydia trachomatis, Mycoplasma genitalium, Mycoplasma hominis, Neisseria gonorrhoeae, Ureaplasma parvum* and *Ureaplasma urealyticum* and the parasite *Trichomonas vaginalis. Human Papilloma Virus* with subtyping was tested for on a separate platform.
5. Three PCR controls were included for each sample, PCR Positive Control, and probes for both pan bacteria and GAPDH.

### Microbial DNA extraction (mDNA)

Vaginal swabs, semen or FCU deposits had microbial DNA (mDNA) only extracted using the QIAmp^®^ UCP Pathogen Minikit #50,214 (Qiagen) [26] according to the manufacturer’s instructions.

### Testing platform for molecular testing of microbial species

The 96-well microtitre plates were produced in 24 × 4 samples format. Microbial qPCR mastermix Format A with Rox (a passive reference dye) was used containing PCR primers that detected the bacterial 16S rRNA gene. The amplified product was detected using target-specific fluorescent hydrolysis probes present in designated wells.

### qPCR set up

A standardised amount of 125 ng of mDNA was recommended and used per reaction. This constant input of mDNA allowed for comparison of results.

A total volume of 25 µL reaction mix was added per well. qPCR was performed on the Quant Studio 6 Flex, Applied Biosystems platform (Life Technologies).

Thermocycling conditions: activation 10 min 95 °C 1 cycle, 2-step cycling of 45 cycles—denaturation 15 s 95 °C, annealing and extension 2 min 60 °C.

The threshold cycle (Ct) was calculated for each well for data analysis.

#### Molecular testing for HPV

The EUROIMMUN HPV testing platform detected 30 genital HPV types Table 2, (EUROIMMUN, Perkin Elmer) [13].

**Table 2:** HPV types tested using the EUROIMMUN array testing platform

HPV subtypes detected	
18 high-risk HPV	12 low-risk HPV
16	6
18	11
26	40
31	42
33	43
35	44
39	54
45	61
51	70
52	72
53	81
56	89
58	
59	
66	
68	
73	
82	

### Viral DNA extraction

Viral DNA was extracted from vaginal swabs using the QIAmp DNA Mini Protocol #51304 (Qiagen) [24] according to the manufacturer’s instructions.

Viral DNA was extracted from semen and FCU deposits using the High Pure PCR Template Preparation Kit #11796828001 (Roche) [27] according to the manufacturer’s instructions.

### qPCR setup

The qPCR was set up according to the manufacturer’s instructions of product number MN 2540-2005 (EUROIMMUN, Perkin Elmer). The Microarray platform detected oncogenes E6/E7 [13] using subtype specific primers and probes.

#### Culture for the detection of *Mycoplasma hominis* and *Ureaplasma urealyticum* (and *Ureaplasma parvum*)

Semen samples were cultured using the Mycoplasma Duo Test #62740 (Bio-Rad) according to the manufacturer’s instructions [25].

It should be noted that according to the manufacturer’s instructions, the Mycoplasma Duo kit can also detect *Ureaplasma parvum* in the *U. urealyticum* well. *U. parvum* was originally a Biovar of *U. urealyticum* before it was proposed to be renamed as a distinct species based on phylogenetic analysis [25, 28].

### Data analysis

#### Quantitative levels of microbial species detected

The presence of a particular microbial species was determined using the crossing point (Ct) where a positive signal had crossed the baseline. Samples that crossed the baseline at 20–22 cycles were awarded a value of 10, compared to a sample which required 38–40 cycles for detection which were awarded a value of 1.

#### Qualitative analysis of the percent of samples that were positive

The data were converted to being positive (+) or negative (−) for each microorganism. The percent of samples with a positive signal was estimated.

From these data, a one tailed Z proportionality test was used to detect the differences between the study groups where a *p* value of < 0.05 was considered significant. Samples collected at the same time were compared—Samples A + (implantation) were compared to Sample A− (no implantation), etc.

#### Data analysis of testing for HPV

Analysis and interpretation was fully automated using EUROArrayScan software [13].

## Results

Thirty couples were retrospectively categorised into two groups, those who achieved implantation (implantation only, implantation and pregnancy only or a live birth) *n* = 15 and those who did not achieve implantation *n* = 15.

### Nugent Gram stain scores obtained from vaginal swabs

Gram staining indicated there was minimal difference in Nugent scores from vaginal swabs taken at baseline (A) and ET (B), from women who achieved implantation and those who did not (Table 3). In fact, most Nugent scores tended to be predominantly anaerobic from both groups of women.

**Table 3:** Nugent scores from vaginal swabs from women sampled at the baseline cycle (Sample A) and at embryo transfer (Sample B) for those who achieved implantation and those who did not

	Women who achieved implantation Nugent score mean	Women who achieved implantation with a predominance of *Lactobacilli* detected by qPCR	Women who achieved implantation with a predominance of anaerobes detected by qPCR	Women who did not achieve implantation Nugent score mean	Women who did not achieve implantation with a predominance of *Lactobacilli* detected by qPCR	Women who did not achieve implantation with a predominance of anaerobes detected by qPCR
Sample A baseline vaginal swab	2.6	33%	40%	2.7	33%	27%
Sample B embryo transfer vaginal swab	2.9	7%	47%	3.1	13%	40%

The percent of samples that were positive for *Lactobacillus* spp. and anaerobes detected by qPCR is added for comparison.

In women who had a Nugent score indicating anaerobes, none of the 4 urogenital anaerobes tested for was found in 4 of 15 women who achieved implantation and 2 of 15 women who did not. The observation suggested that the anaerobes seen microscopically were not included in the customised qPCR testing panel.

Three further distinctive anaerobes *Mobiluncus *sp*.*, *Peptostreptococcus *sp*.* and *Atopobium vaginae* implicated in anaerobic vaginosis were not included in the qPCR panel as they can be easily identified by their specific morphology in the Gram stain, and these were not seen.

### Molecular testing for microbial species

qPCR methodology for microbial species detected organisms with increased sensitivity compared to traditional microscopy. Table 3 demonstrates a comparison of molecular detection to a Nugent score.

Table 3 also indicates that for Sample A only a third of women in both groups (implantation or no implantation) had a predominant population of *Lactobacillus *spp. in baseline samples, dropping to just one or two samples for Sample B from both groups of women.

It was noted that the STIs *Chlamydia trachomatis* and *Neisseria gonorrhoeae* and potential urogenital pathogens *Enterococcus faecium* and *Streptococcus pyogenes* were not detected in any samples by qPCR. Therefore, these bacteria were excluded from the data analysis.

The most commonly detected bacterium in both groups was *Lactobacilli crispatus*. It was more common for a woman to have the same microorganism detected or not detected in samples A and B. Thus, *levels* of microbial species detected altered, but not their *presence*.

#### Quantitative levels of microbial species detected

Samples from the two groups of couples were quantitatively compared using the students *t* test.

### Individual samples

- In Samples A and B, there was no significant difference between the two groups in the means for each microbial species.
- Sample C, only *Lactobacilli crispatus* was significantly different in the male partners of women who did not achieve implantation *p* = 0.04.

### Dynamic characteristics of the microbiome

iii. In women who achieved implantation, the levels of microbial species were determined—Samples A vs B were compared to determine the variation in microbial flora. Anaerobe *Finegoldia magna p* = 0.02 had significantly different levels higher in Sample A.
iv. In women who did not achieve implantation—Samples A vs B were compared. Anaerobes *Anaerococcus prevotii* *p* = 0.05 and *Finegoldia magna p* = 2 × 10^–4^ had significantly different levels higher in Sample A.
v. In couples who achieved implantation–baseline Samples A vs C were compared. Anaerobes *Anaerococcus prevotii p* = 0.03, *Finegoldia magna p* = 7 × 10^–4^ and *Prevotella bivia* *p* = 6 × 10^–4^, and *Lactobacilli jensenii p* = 8 × 10^–5^ and *Lactobacilli gasseri p* = 0.03 had significantly different levels which were higher in Sample A.
vi. In couples who did not achieve implantation,—baseline Samples A vs C were compared. Anaerobes *Anaerococcus prevotii p* = 9 × 10^–5^ and *Finegoldia magna p* = 1 × 10^–4^, and *Lactobacilli gasseri p* = 0.001 and *Lactobacilli iners p* = 0.03 had significantly different levels which were higher in Sample A.

The results in paragraphs (iii) and (vi) led us to believe that a woman self-sampling (Sample A) possibly had a more effective sampling style for obtaining vaginal epithelial cells to which microbial species often adhere. Despite *levels* of microbial species detected being increased, this did not often correlate with the success or failure of implantation. Using quantitative levels revealed little useful information.

#### Qualitative analysis of the percent of samples that were positive

Thus, alternative analysis was required. Samples from the two groups of couples were qualitatively compared using the *Z* test.

**Fig. 1:**
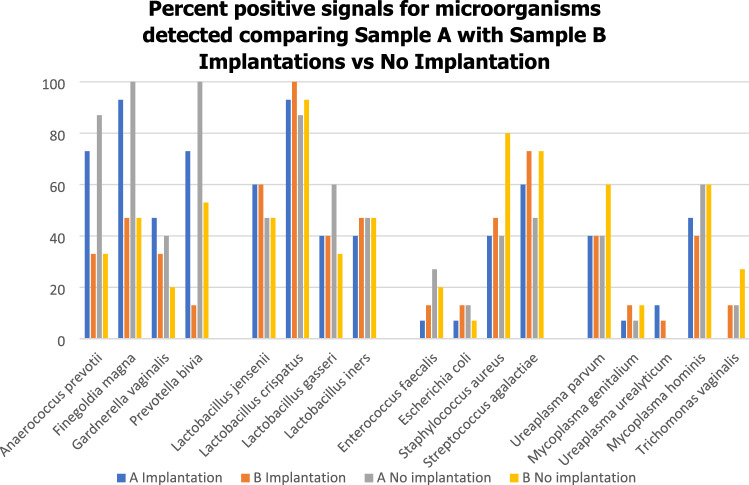
The percent of samples that were positive for each microorganism in women for Samples A and B in those who achieved implantation and those women who did not

**Fig. 2:**
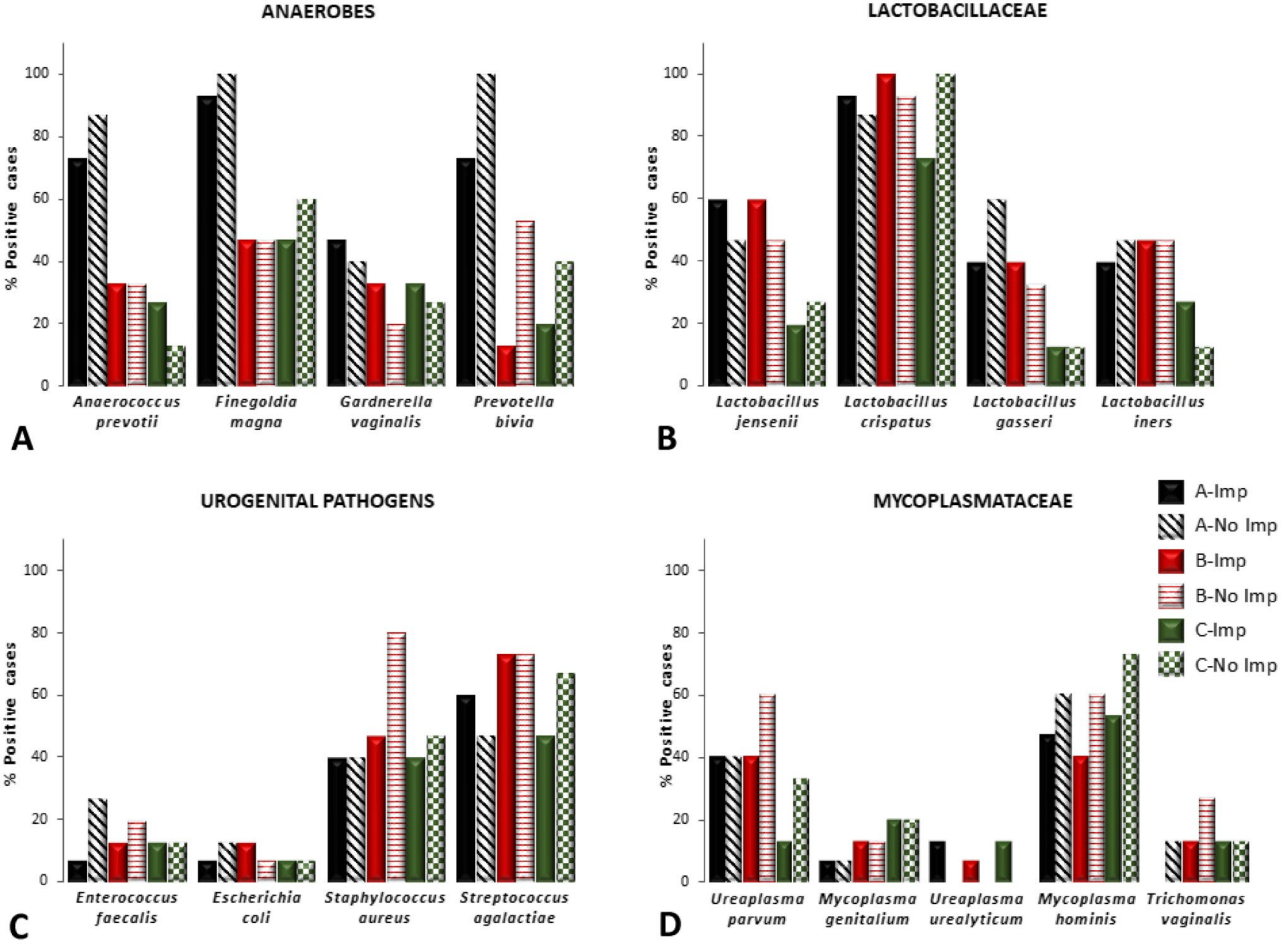
**A**–**D** The percent of samples that were positive for four groups of microbial species in couples who achieved implantation (**A** Imp, **B** Imp and **C** Imp) and those who did not (**A** No Imp, **B** No Imp, **C** No Imp). Solid bars are for those couples who achieved implantation and hatched bars are for couples who did not achieve implantation

- The percent of positive signals were determined in women from both groups with Samples A vs B (Fig. 1). A reduction in some anaerobic numbers in Sample B in both groups of women was noted whereas for *Lactobacilli *sp., potential urogenital pathogens and STIs the levels detected remained stable demonstrating stability of some urogenital flora during two luteal phases of the cycle [29]. Of the anaerobes tested, the percent of samples that were positive for *Anaerococcus prevotii, Finegoldia magna* and *Prevotella bivia* were more unstable between samples. Substantially more microbial species were detected in the baseline Sample A than in Sample B at ET. Interestingly, *Gardnerella vaginalis* levels tended to remain stable, possibly indicating that this bacterium did not influence the success or failure of implantation (Fig. 1). Further, *Gardnerella vaginalis* was the least commonly detected anaerobe. Variable signals for anaerobes only, indicated that their presence was not an artefact.
- The percent of samples that were positive for all 3 sample sets were then compared and presented within separate groups of microbial species—anaerobes, Lactobacillaceae, potential urogenital pathogens and Mycoplasmataceae (including *Trichomonas vaginalis*) (Fig. 2A–D). Figure 2 indicates the high number of samples that were positive for *Lactobacilli crispatus* and the moderate number of samples positive for *Lactobacilli iners* in both implantation and non-implantation groups indicating that these bacteria do not influence success or failure of implantation.
- The percent of samples that were positive were then observed within each sample set where Sample A implantation denoted as A + was compared to Sample A no implantation denoted as A- and so on (i.e. B + vs B − ; C + vs C − ). Table 4 demonstrates where the Z test detected some significant differences between the study groups.

**Table 4:**
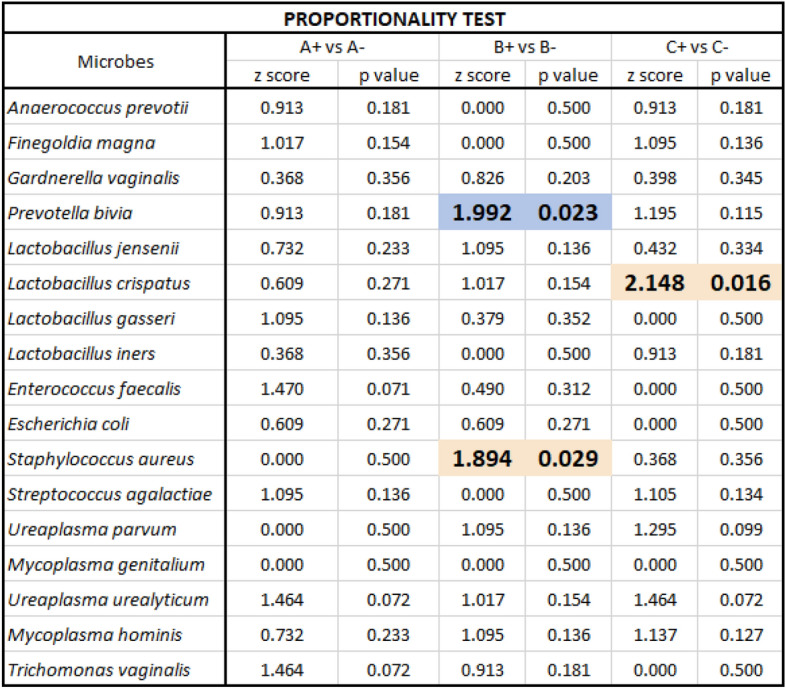
The Z score and *p* values of microbial species present in individual samples (A, B and C) of 15 couples who did achieve implantation (+) and 15 couples who did not (−)

Comparison of Sample B in women who achieved implantation denoted B + and Sample B in women who did not achieve implantation denoted B −  produced a *p* value that was significant for *Prevotella bivia* and *Staphylococcus aureus.* The percent of samples that were positive were significantly higher in women who did not achieve implantation. Sample C produced a significant *p* value for *Lactobacillus crispatus* where levels were higher for male partners of women who did not achieve implantation*.*

### Molecular testing for *Human Papilloma Virus* species with subtyping on a separate platform

HPV was detected in 5 women and 1 male of the total 30 couples, although there was an absence of HPV in their partners. In the group who achieved implantation—of three women, one woman had a low-risk subtype, another woman had 3 subtypes detected of which one was high risk, a further woman had a high-risk subtype detected, while another male partner also had a high-risk subtype of HPV detected. Of the two women with HPV detected who did not achieve implantation, one had a high-risk subtype and the other a low-risk subtype detected. We were unable to detect any HPV DNA in one woman who achieved a live birth.

### Culture for the detection of *Mycoplasma hominis*,* Ureaplasma urealyticum* (and *Ureaplasma parvum*)

The potential influence of the male partner was further examined, whereby a semen sample from the male partner taken at ET was cultured for *Mycoplasma* spp.

For women who achieved implantation, their partners’ samples were positive by culture for *Ureaplasma urealyticum* in 4 cases, none was confirmed by qPCR. For women who did not achieve implantation their partners displaying a positive result in 4 cases, 3 were confirmed by qPCR. qPCR detected *Mycoplasma* species with increased sensitivity in 13 couples who achieved implantation and in 15 couples who did not. Dual infections were commonly detected by qPCR.

## Discussion

This project studies the microbiome of the urogenital tract in IVF couples. These sites have the potential to influence the conditions for implantation in the uterine cavity.

Although this pilot study where the numbers recruited are limited, this is within the common range of other IVF studies [12, 29–32]. We investigated ‘if a panel of individual microbial species are able to predict implantation success during IVF’ and we present these preliminary data.

The aim of this study was to determine a foundation for an accessible, predictive test of the urogenital microbiome profiling to fit into the routine IVF workup. We used a collection method easily accomplished by both patient and clinician.

We found that using the traditional methodology of Nugent scores was of little assistance as a pronostic tool to indicate implantation outcome. Most vaginal samples from either group (i.e. implantation or no implantation) exhibited a predominance of anaerobes, only a few samples were dominated by *Lactobacilli *sp*.* This technique is still used to ascertain vaginal flora [4]. The lack of value of the Nugent scoring in the context of our current study motivated us to use the qPCR technique.

The procedure for Microbial DNA qPCR Assay Kits is simple and can be performed in any molecular laboratory with a real-time PCR instrument. Thus, this approach is easily achievable, within the range of an IVF budget and providing a timely result that can be clinically applicable.

First, we estimated the quantitative *levels* of microbial species present, and second, the qualitative presence or absence of a microbial species.

### Quantitative levels of microbial species detection in sample A for women who achieved implantation are denoted A + vs sample A for women who did not achieve implantation are denoted A −,  and samples B + vs B − 

Significant differences of some anaerobes and some *Lactobacilli *sp*.* in both groups were found. It was noted that in individual women the level of microbial species in Sample A often varied from that of Sample B supporting the suggestion that a dynamic environment is involved [21]. Of particular note is our observation that anaerobes were the main driver of bacterial dynamics. This observation questions why different bacteria behave in a different manner and how the dynamics of the microbiome [19–21] are regulated.

Further, potential urogenital pathogens, *Staphylococcus aureus* and *Streptococcus agalactiae* and STIs *Mycoplasma hominis* tended to be commonly detected in both groups (Fig. 1). The observation possibly suggesting that a degree of microbial diversity is required to maintain basic physiological function of the urogenital tract.

### Quantitative levels of microbial species detection in sample C for men whose partners achieved implantation are denoted C + vs sample C for men whose partners did not achieve implantation are denoted C − 

Only one *Lactobacilli *sp*.* was significantly higher in males of women who did not achieve implantation. Interestingly, this finding is not supported by the observation of their female partners.

Further, the quantitative *level* of microbial pathogens detected was very reliant on sample collection. Interestingly, it appeared that woman may have had a more vigorous sampling style as found in Sample A, than clinicians sampling Sample B, Sample A contained increased numbers of vaginal epithelial cells to which microbial species often adhere. Possibly, other external activities such as douching or increased sexual intercourse in the IVF cycle may have also contributed to a lower bacterial yield in Sample B.

Standardised sampling in accessing vaginal epithelial cells is not applicable. Frequently, when Sample B was collected by the clinician, more mDNA had to be loaded to reach the same concentration of 125 ng for testing. This factor contributed to us simplifying the analysis of data to a qualitative analysis. Thus, the difference in sampling quality had no effect on the obtained results.

### Qualitative analysis of qPCR results

The findings were usually similar when comparing the microbial presence or absence between couples who achieved implantation and those who did not. Samples notably generally had the same microbial species detected either by both samples being positive or negative, with minimal variation; only a few samples differed. These results indicated there was not rapid or frequent alteration of the qualitative nature of the microbiome in these couples.

The percent of samples that were positive suggests that the mere presence of a microorganism had an effect on embryo implantation. A number of individuals who achieved implantation nurtured a level of anaerobes in the urogenital tract commonly reported to be associated with non-implantation. However, the Z test produced two significant *p* values in Sample B, in women who did not achieve implantation. Increased levels of the anaerobe *Prevotella bivia,* as confirmed by others [2, 7–10] and *Staphylococcus aureus* were detected. The lack of significant detection of other selected anaerobes such as *Gardnerella vaginalis* does not support these reported findings. Possibly, some anaerobes chosen to be included in the testing panel may not have been as active as others. Pro-inflammatory characteristics present in anaerobic/bacterial vaginosis [18, 33] as well as potential urogenital pathogens can assist implantation [34]. Our finding indicates that while a proportion of ‘healthy’ microbial species may be important, rates of implantation may be dependent on the biodiversity of microbial species. The implication of such a conclusion is that the interactions and co-effects of one microorganism on another must be delineated in the context of the efficient application of IVF. This unique, custom array testing panel allows the option to choose new combinations of further microbial species.

Another significant finding of *Staphylococcus aureus* also in Sample B of women who did not achieve implantation could be explained by sampling technique. However, this trend was not noted in Sample B from women who did achieve implantation. *Staphylococcus aureus* is considered as a skin contaminant. There were no significant differences detected for microbial species in Sample A in women who achieved implantation and those who did not. Sample C also had a significant *p* value detected for one *Lactobacilli* sp.in males of couples who did not achieve implantation.

The lack of HPV detection may be explained where negative partners may have resolved their HPV infection either by vaccination or development of their own immunity [35]. The incidence of HPV detected was too low to ascribe any influence to implantation outcome and is not a robust predictor in our sample population.

The vaginal cavity has the capability to fluctuate creating a dynamic urogenital microbiome environment [36–38] as observed in this work, and is considered to be a likely influence in the success of implantation rates [39, 40].

Further, an increase in microbial populations detected in women only, had no effect on implantation outcome. Yet, couples were advised to have sexual intercourse as frequently as possible in the IVF cycle, where it would be expected that both couples would share similar microbial flora. This finding was possibly influenced by the female urogenital tract being more hospitable to microbial species compared to the male who may or may not have harboured a different or less hospitable environment [41]. Further, with less squamous epithelial cells obtained from the male urine sample this may have been a contributing factor to a reduced associated presence of microbial flora. Indeed, in the male samples there were reduced levels of some anaerobes and some *Lactobacillus *spp*.* compared to the female partner These organisms may have been more sensitive to their environment than other bacteria which remained at similar levels as the female counterpart such as *Gardnerella vaginalis*, *Lactobacilli crispatus*, potential urogenital pathogens and the *Mycoplasma* spp.

Male reproductive proteins (MRPs) can also have broad implications for successful reproduction. MRPs have the potential to influence the composition of the vaginal microbiome and thus the success of implantation [42]. But in this study, a wide variation in the male urogenital microbiome often did not always correspond with the women’s vaginal microbiome.

## Conclusion

The results of this study challenges a concept of current thinking and is at the interface of research and clinical application. The unique methodology of this pilot project is most suitable as a foundation on which to develop an affordable, timely test of microbiome profiling in the routine IVF workup. Using the two indicators that were detected to have a significant influence, these results can be extrapolated to a rapid antigen test for a woman to self-sample prior to ET as an indicator of microbial species present which could influence  implantation outcome. The addition of further microbial targets (yet to be determined) can also be combined in this predictive test for vaginal preparedness on the day of ET.

## Data Availability

Not applicable.
